# Ball games and nutrition counseling improve postural control in overweight children

**DOI:** 10.1186/s12887-015-0523-4

**Published:** 2015-12-11

**Authors:** Benita Kuni, Nina Elisabeth Rühling, Ulrike Hegar, Christina Roth, Holger Schmitt

**Affiliations:** Clinic for Orthopedics and Trauma Surgery, Center for Orthopedics, Trauma Surgery and Spinal Cord Injury Heidelberg, University Hospital, Schlierbacher Landstr. 200a, 69118 Heidelberg, Germany; Institute of Sports and Sports Sciences, University of Heidelberg, Im Neuenheimer Feld 720, 69120 Heidelberg, Germany

**Keywords:** Pediatric overweight, Pediatric obesity, One legged standing test, Ball School, Food

## Abstract

**Background:**

Motor skills are impaired in overweight children whose levels of physical activity are low and these children are more likely to sustain lower extremity injuries.

The purpose of this study was to analyze prospectively in overweight children the influence of ball games and nutrition counseling on postural control.

**Methods:**

In all, 46 overweight children (age: 6–12 years, BMI: female: 25.2 ± 3.6 kg/m^2^, male: 26.2 ± 2.8 kg/m^2^ (mean value ± standard deviation) were examined prospectively in four randomized groups (ball games, nutrition counseling, ball games and nutrition counseling, and group without intervention) for six months. A one-legged standing test was performed.

**Results:**

The children demonstrated improved postural control after six months of intervention: mean difference M1-M2 ± standard deviation: 5 ± 6 error points (*p* < 0.001, T = 4.906), whereas the control group without intervention did not show any significant improvement: 2 ± 8 error points (*p* = 0.357, T = 0.972).

**Conclusions:**

Ball games and nutrition counseling have a positive influence on postural control and therefore could help prevent injury.

**Trial registration:**

ClinicalTrials.gov Identifier: NCT01825174.

Registered April 2, 2013

## Background

Excess weight impairs postural control [1, 2], which can already occur early in childhood [3, 4]. Motor skills are impaired in overweight children with a low level of physical activity, as tested by means of the body coordination test for children (BCC) [5, 6].

Obese children are significantly more likely to sustain lower extremity injuries than upper extremity injuries (as compared to nonobese children) [7] and they show a higher incidence of extremity fractures [8, 9]. Indeed, strategies for preventing lower extremity injuries among obese children should be multidisciplinary in design, involving nutritionists, sports trainers, and general health care providers [10]. Intervention studies with adolescent athletes have shown that improving postural control helps to achieve lower injury rates when compliance to the program is high [11].

Overweight children might not join sports activities with the same ease as nonoverweight children. The initial idea of this study was to use the preexisting structure of the “Ball School Heidelberg” [12], an educational program for introducing children to all sorts of ball activities and games, and to adapt this program to the special group of overweight children [13]. This increase in physical activity was meant to be controlled by comparing it with other interventions and with a noninterventional control arm.

The aim of this study was to observe prospectively over six months the impact of ball games and nutrition counseling on postural control. The history of injuries was prospectively recorded.

Our main hypothesis was that postural control would improve with intervention (ball games and nutrition counseling).

## Methods

“Ball school – easy” is an interdisciplinary project of the Heidelberg University Hospital. The aim of this major project was to extend the offer of the program “Ball School Heidelberg” (which is a basic introduction to ball games for school children [12]) to the specific population of overweight children [13].

Overweight children were recruited via press releases. These children (*n* = 46, f(emale): 8 y.(ears) 9 mo.(nths) ± 1 y. 2 mo., BMI 25.2 ± 3.6 kg/m^2^, perc(entiles) 97.7 ± 3.0 %, m(ale): 9 y. 6 mo. ± 1 y. 4 mo., 26.2 ± 2.8 kg/m^2^, perc.: 98.2 ± 2.5 % (mean value ± standard deviation)) were examined twice: before the intervention (Measurement 1, M1) and after six months (M2). Choosing this intervention period, we were able to accomplish all the measurements in the time span of one school year. Thus, dropout due to school changes/moving was minimized. Six months also seemed to represent a comparable time frame with respect to other school-based intervention studies [14, 15].

Children were determined to be overweight (inclusion criteria) when their BMI was greater than the gender-, race-, and age-specific 90^th^ percentile from the National Children Health and Nutrition Examination Survey [16, 17]*.* Among the participants, 3 girls and 2 boys were overweight; all the others were obese. In our data presentation, the term “overweight children” is used for both overweight and obese children. The children were randomized by lot into four groups, three with different interventions and one control group without any intervention. A compliance rate of a minimum of 70 % participation in the ball games and 6/9 nutrition sessions was required; otherwise the child was excluded. In detail the program involved: for group A: 90-min ball games class twice weekly (*n* = 11 at M1, *n* = 7 at M2; 3 excluded due to compliance, one dropped out for other reasons; the compliance rate of the remaining group (cr) = 84 ± 8 %); group B: 9 units of nutrition counseling (*n* = 13, 12; cr = 80 ± 13 %); group C: ball games and nutrition counseling (*n* = 12, 11; one dropped out for other reasons, cr ball = 84 ± 9 %, cr nutrition = 81 ± 7 %), and the control group without intervention (*n* = 10, 10). The dropouts for other reasons were mostly personal (school change/moving/not showing up for the measurement). All but a few sessions were conducted by the same sports therapist.

The ball games were structured in order to teach basic tactical elements, coordinative ball skills, and technique (ball path/timing/control, teammate’s/opponent’s behavior).

In detail this included: elementary game techniques (throwing, catching), mostly low level exercises (to promote the feeling of enhancing achievement), exercises without a ball (catch, coordination exercises), and psychosocial games were part of the program. The children received stickers at the end of the class when their participation and behavior were adequate (then for 10 stickers a gift).

The nutrition counseling was organized as three evenings (1.5 h(ours) each) with the parents (knowledge transfer), three sessions with the children (1.5 h each; experience in eating and drinking), and three activities together (grocery shopping, fast food/restaurant, and closing party). A supplementary email hotline was also available.

In detail, the program was composed as shown in Table 1.

**Table 1:** Program for the nutrition counseling sessions for parents, children, and parents and children together

Parents	Children	Parents & children
Definition, importance, and consequences of pediatric overweight	Game-related knowledge acquisition	Completing a quiz
Goals of the nutrition therapy	Role games	Joint fictive food shopping in the family team
Nutrition pyramid	Nutrition pyramid	Meal plan
Educational and nutritional errors	Difference between hunger and appetite	Stock check
Basics for pediatric nutrition	Portions	Making a list
Food shopping		Food shopping
Meal structure, composition, and amount		Checking the basket
Fast food	Tests for taste and senses	Preparing tasty snacks
Information about how to motivate children		
Family resources		
Exceptional situations		
Prevention of falling back into poor habits		

All participants were given the opportunity to join ball and nutrition programs after the end of the study. Musculoskeletal pain and injuries were prospectively assessed.

Informed written consent was obtained from the parents and guardians of all children. The independent Ethical Committee of the University of Heidelberg approved this study. This study was performed in accordance with the ethical standards in sports and exercise science research [18, 19].

### One-legged standing test

The postural control was measured by means of a one-legged standing test (OST) on a gymnastic mat (8 cm thick), using the error point system of the BESS score (Balance-Error Scoring System) [20, 21]. The test person stood on their dominant foot (the preferred foot for kicking a ball). The free leg had to hang slightly bent, freely, so that there was approximately 10 cm of space between foot tip and mat. The arms were placed on the iliac crests. The test was performed one minute with eyes open and, after a one-minute break, with closed eyes. Error points were: putting down the free leg, abducting or bending the free leg more than 30°, changing the arm position, hopping or raising the metatarsal or the heel, and peeking.

### Statistical analysis

The statistical analysis was performed with Microsoft Excel (Microsoft Incorporation) and SPSS (SPSS Inc., Chicago, Ill, USA). Measures of central tendency and dispersion were calculated for all variables, and normal distribution was assessed by using the KS Test. The comparisons were calculated by using the Student´s *t*-test for normally distributed data and by nonparametric tests for non-normally distributed data (Wilcoxon for dependent samples). Correlations were calculated by means of the Pearson’s correlation coefficient.

Due to the observational nature of the study, the significance level was assigned at 5 % for all comparisons.

## Results

Between M1 and M2, postural control (error points of the BESS score) with open eyes improved in all three intervention groups: in the ball games group (mean difference M1-M2 ± standard deviation: 5.6 ± 3.5 error points, T = 5.264, *p* = 0.005), in the nutrition counseling group (6.0 ± 6.3 error points, T = 3.324, *p* = 0.007), and, with a tendency, in the group with ball games combined with nutrition counseling (3.7 ± 6.3 error points, T = 1.969, *p* = 0.077). With closed eyes, no significant changes were found.

Between M1 and M2, the most common injuries were ankle sprains (8/40); furthermore, one knee and three finger sprains were reported. At M2, 10 of the 46 overweight children reported musculoskeletal pain (lower extremities, upper extremities, or back pain).

## Discussion

The purpose of this study was to analyze prospectively the influence of ball games and nutrition counseling on postural control in overweight children.

Both interventions showed significant positive effects on postural control in our study population.

### Postural control

Postural control is impaired in healthy young men when extra weight is added at the upper part of the trunk [22]. In overweight children, mediolateral sway is higher than in nonoverweight children [4]. Overweight prepubertal boys display a lower capacity in static and dynamic postural skills [1].

Fine motor skill performance is impaired in overweight children while standing on a balance beam [5]. Not only postural control, but also ball skills were found to be significantly better in nonoverweight children as compared with obese ones [23]. Obese children might also suffer from underlying perceptual-motor coordination difficulties [5].

Postural control improved with both interventions. Even nonspecific training of postural control (as a collateral effect of the ball school training) showed a positive influence. The BESS score is commonly used for assessing postural control, also in children [21, 24]. An important learning effect [24] is excluded since the control group without intervention did not improve significantly.

### Ball School intervention and nutrition counseling

Ball games were found to be beneficial in developing balance [25]. Initially, the program “Ball School Heidelberg” was established in 1998 for introducing school children to different ball games, to develop basic motor skills and coordination. Since this program was in place, the training programs and the trainers were already prepared. The study group chose this program structure as one of the intervention arms of this study. The aim was to give overweight children access to this program and, in the future, make it easier to join any other sports training offer and thus facilitate the integration of overweight children in sports groups. Even at a young age, we found a higher percentage of overweight children who did not participate in any sports other than school sports.

Weight reduction in adults has been shown to improve postural control [26]. In overweight adults, weight loss improves postural control more efficiently than increasing muscle strength [27]. Previously, activity plus diet programs were efficacious in improving overweight children’s movement skill proficiency [28].

The fact that both interventions, ball training and nutrition counseling, were effective in improving the postural control could indicate a nonspecific maturation process as a bias. However, postural control did not improve in the control group without any intervention, which excludes the maturation bias. In addition, we believe that the training and developing a concept for changing nutrition patterns can influence the children’s body awareness and, therefore, their postural control.

### Injuries

Prospectively, ankle sprains were the most common injuries. It is possible that additional weight combined with postural control deficits in overweight children influences the rate of lower extremity injuries [7, 8].

The consequences of deviations from normal physiology, such as increased risk of musculoskeletal injuries, may be alleviated by interventions aimed at improving muscle strength, coordination, and postural control and by nutrition counseling. Limitations of this study include the relatively small sample size and relatively short intervention period compared to other settings [29, 30].

## Conclusions

Overweight children could benefit, in terms of postural control, from a multidisciplinary intervention program.

## References

[1] Deforche BI, Hills AP, Worringham CJ, Davies PS, Murphy AJ, Bouckaert JJ (2009). Balance and postural skills in normal-weight and overweight prepubertal boys. Int J Pediatr Obes.

[2] Hue O, Simoneau M, Marcotte J, Berrigan F, Dore J, Marceau P (2007). Body weight is a strong predictor of postural stability. Gait Posture.

[3] Goulding A, Jones IE, Taylor RW, Piggot JM, Taylor D (2003). Dynamic and static tests of balance and postural sway in boys: effects of previous wrist bone fractures and high adiposity. Gait Posture.

[4] McGraw B, McClenaghan BA, Williams HG, Dickerson J, Ward DS (2000). Gait and postural stability in obese and nonobese prepubertal boys. Arch Phys Med Rehabil.

[5] D’Hondt E, Deforche B, De Bourdeaudhuij I, Lenoir M (2008). Childhood obesity affects fine motor skill performance under different postural constraints. Neurosci Lett.

[6] Graf C, Koch B, Kretschmann-Kandel E, Falkowski G, Christ H, Coburger S (2004). Correlation between BMI, leisure habits and motor abilities in childhood (CHILT-project). Int J Obes Relat Metab Disord.

[7] Pomerantz WJ, Timm NL, Gittelman MA (2010). Injury patterns in obese versus nonobese children presenting to a pediatric emergency department. Pediatrics.

[8] Rana AR, Michalsky MP, Teich S, Groner JI, Caniano DA, Schuster DP (2009). Childhood obesity: a risk factor for injuries observed at a level-1 trauma center. J Pediatr Surg.

[9] Valerio G, Galle F, Mancusi C, Di Onofrio V, Guida P, Tramontano A (2012). Prevalence of overweight in children with bone fractures: a case control study. BMC Pediatr.

[10] Barton M (2010). Screening for obesity in children and adolescents: US Preventive Services Task Force recommendation statement. Pediatrics.

[11] Steffen K, Emery CA, Romiti M, Kang J, Bizzini M, Dvorak J (2013). High adherence to a neuromuscular injury prevention programme (FIFA 11+) improves functional balance and reduces injury risk in Canadian youth female football players: a cluster randomised trial. Br J Sports Med.

[12] Roth K, Kröger C (2015). Ballschule: ein ABC für Spielanfänger, 5.

[13] Hegar U (2012). Ballschule - leicht gemacht: Auswirkungen eines Ernährungs- und Bewegungsprogramms auf entwicklungsrelevante Parameter bei übergewichtigen und adipösen Kindern.

[14] Chen Y, Ma L, Ma Y, Wang H, Luo J, Zhang X (2015). A national school-based health lifestyles interventions among Chinese children and adolescents against obesity: rationale, design and methodology of a randomized controlled trial in China. BMC Public Health.

[15] Dobbins M, Husson H, DeCorby K, LaRocca RL (2013). School-based physical activity programs for promoting physical activity and fitness in children and adolescents aged 6 to 18. Cochrane Database Syst Rev.

[16] Kromeyer-Hauschild K, Wabitsch M, Kunze D, Geller F, Geiß HC, Hesse V (2001). Perzentile für den body-mass-index für das Kindes- und Jugendalter unter Heranziehung verschiedener deutscher Stichproben. Monatsschr Kinderheilkd.

[17] Cole TJ, Bellizzi MC, Flegal KM, Dietz WH (2000). Establishing a standard definition for child overweight and obesity worldwide: international survey. BMJ.

[18] Harriss DJ, Atkinson G (2011). Update--Ethical standards in sport and exercise science research. Int J Sports Med.

[19] Harriss DJ, Atkinson G (2009). International journal of sports medicine - ethical standards in sport and exercise science research. Int J Sports Med.

[20] Riemann BL, Guskiewicz KM (2000). Effects of mild head injury on postural stability as measured through clinical balance testing. J Athl Train.

[21] Steib S, Zech A, Hentschke C, Pfeifer K (2013). Fatigue-induced alterations of static and dynamic postural control in athletes with a history of ankle sprain. J Athl Train.

[22] Ledin T, Odkvist LM (1993). Effects of increased inertial load in dynamic and randomized perturbed posturography. Acta Otolaryngol.

[23] D’Hondt E, Deforche B, De Bourdeaudhuij I, Lenoir M (2009). Relationship between motor skill and body mass index in 5- to 10-year-old children. Adapt Phys Activ Q.

[24] Valovich McLeod TC, Perrin DH, Guskiewicz KM, Shultz SJ, Diamond R, Gansneder BM (2004). Serial administration of clinical concussion assessments and learning effects in healthy young athletes. Clin J Sport Med.

[25] Yazicioglu K, Taskaynatan MA, Guzelkucuk U, Tugcu I (2007). Effect of playing football (soccer) on balance, strength, and quality of life in unilateral below-knee amputees. Am J Phys Med Rehabil.

[26] Maffiuletti NA, Agosti F, Proietti M, Riva D, Resnik M, Lafortuna CL (2005). Postural instability of extremely obese individuals improves after a body weight reduction program entailing specific balance training. J Endocrinol Invest.

[27] Handrigan G, Hue O, Simoneau M, Corbeil P, Marceau P, Marceau S (2010). Weight loss and muscular strength affect static balance control. Int J Obes (Lond).

[28] Cliff DP, Okely AD, Morgan PJ, Steele JR, Jones RA, Colyvas K (2011). Movement skills and physical activity in obese children: randomized controlled trial. Med Sci Sports Exerc.

[29] Roth K, Mauer S, Obinger M, Ruf KC, Graf C, Kriemler S (2010). Prevention through activity in Kindergarten Trial (PAKT): a cluster randomised controlled trial to assess the effects of an activity intervention in preschool children. BMC Public Health.

[30] Telford RD, Cunningham RB, Fitzgerald R, Olive LS, Prosser L, Jiang X (2012). Physical education, obesity, and academic achievement: a 2-year longitudinal investigation of Australian elementary school children. Am J Public Health.

